# Safety and efficacy of stem cell therapy for Crohn’s disease: an umbrella review of systematic reviews

**DOI:** 10.1097/JS9.0000000000002104

**Published:** 2024-09-30

**Authors:** Lakshmi Thangavelu, Syam Mohan, Hassan A. Alfaifi, Abdullah Farasani, Soumya V. Menon, Pooja Bansal, Chhavi Choudhary, M Ravi Kumar, Raghav Vashishth, Afrah M.A. Al- Rihaymee, Sarvesh Rustagi, Anil K. Malhotra, Muhammed Shabil, Mahalaqua N. Khatib, Quazi S. Zahiruddin, Siddig I. Abdelwahab, Ganesh Bushi, Saleh H.A. Almasabi, Hayam A. Alrasheed, Ali A. Rabaan

**Affiliations:** a Center for Global health Research, Saveetha Medical College and Hospitals, Saveetha Institute of Medical and Technical Sciences, Saveetha University, India; b Substance Abuse and Toxicology Research Centre, Jazan University, Jazan, Saudi Arabia; c School of Health Sciences, University of Petroleum and Energy Studies, Dehradun, Uttarakhand, India; d Pharmaceutical Care Administration (Jeddah Second Health Cluster), Ministry of Health, Saudi Arabia; e Department of Medical Laboratory Technology, Faculty of Applied Medical Sciences, Jazan University, Jazan, Saudi Arabia; f Department of Chemistry and Biochemistry, School of Sciences, JAIN (Deemed to be University), Bangalore, Karnataka, India; g Department of Allied Healthcare and Sciences, Vivekananda Global University, Jaipur, Rajasthan, India; h Chandigarh Pharmacy College, Chandigarh Group of Colleges, Jhanjeri, Mohali, Punjab, India; i Department of Chemistry, Raghu Engineering College, Visakhapatnam, Andhra Pradesh, India; j Department of Surgery, NIMS University, Jaipur, India; k Anesthesia Techniques Department, College of Health and Medical Techniques, Al-Mustaqbal University, Babylon, Iraq; l School of Applied and Life Sciences, Uttaranchal University, Dehradun, Uttarakhand, India; m Graphic Era Institute of Medical Sciences, Graphic Era (Deemed to be University), Clement Town, Dehradun, India; n University Center for Research and Development, Chandigarh University, Mohali, Punjab, India; o Division of Evidence Synthesis, Global Consortium of Public Health and Research, Datta Meghe Institute of Higher Education, Wardha, India; p South Asia Infant Feeding Research Network (SAIFRN), Division of Evidence Synthesis, Global Consortium of Public Health and Research, Datta Meghe Institute of Higher Education, Wardha, India; q Medical Research Centre, Jazan University, Jazan, Saudi Arabia; r School of Pharmaceutical Sciences, Lovely Professional University, Phagwara, India; s Evidence for Policy and Learning, Global Center for Evidence Synthesis, Chandigarh, India; t Department of Clinical Laboratory Sciences, Faculty of Applied Medical Sciences, Najran University, Najran, Saudi Arabia; u Department of Pharmacy Practice, College of Pharmacy, Princess Nourah Bint Abdulrahman University, Riyadh, Saudi Arabia; v Molecular Diagnostic Laboratory, Johns Hopkins Aramco Healthcare, Dhahran, Saudi Arabia; w College of Medicine, Alfaisal University, Riyadh, Saudi Arabia; x Department of Public Health and Nutrition, The University of Haripur, Haripur, Pakistan

**Keywords:** Crohn’s disease, efficacy, meta-analysis, stem cell therapy, systematic review, treatment

## Abstract

**Background:**

Crohn’s disease is a chronic, relapsing form of inflammatory bowel disease marked by severe gastrointestinal inflammation and a broad range of debilitating symptoms. Despite advances in medical treatments, achieving sustained remission remains challenging for many patients. This umbrella review aims to consolidate evidence from various systematic reviews to evaluate the efficacy and safety of stem cell therapies in treating Crohn’s disease.

**Methods:**

This review followed the Joanna Briggs Institute methodology and adhered to PRISMA guidelines. A literature search of PubMed, Web of Science, Embase, and the Cochrane Library covered records up to 20 April 2024. Only systematic reviews and meta-analyses on stem cell therapy for Crohn’s disease were considered. Data were extracted and analyzed for clinical efficacy indicators like remission induction and safety metrics, including adverse events and mortality rates.

**Results:**

Sixteen systematic reviews were included, spanning studies conducted between 2009 and 2023. Stem cell therapy showed a pooled risk ratio (RR) of 1.299 (95% CI: 1.192–1.420) for clinical remission, indicating a 29.9% increased likelihood of remission compared to controls. The pooled RR for healing perianal Crohn’s disease was 1.358 (95% CI: 1.13–1.631), suggesting a 35.8% increased likelihood of healing. A pooled RR of 1.481 (95% CI: 1.036–2.116) shows a 48.1% higher immediate fistula closure rate with stem cell therapy. For long-term outcomes, a RR of 1.422 (95% CI: 1.091–1.854) indicates a 42.2% increased likelihood of maintaining closure. However, stem cell therapy did not significantly impact Crohn’s Disease Activity Index (CDAI) (RR: 1.154, 95% CI: 0.193–6.883) and Perianal Disease Activity Index (PDAI) scores (mean difference at 12 weeks: −0.505, 95% CI: −2.481 to 1.471; mean difference at 24 weeks: −0.338, 95% CI: −1.638 to 0.963). The safety profile was comparable to conventional therapies, with a pooled RR of 0.972 (95% CI: 0.739–1.278) for adverse events and 1.136 (95% CI: 0.821–1.572) for serious adverse events.

**Conclusion:**

Stem cell therapy offers significant progress in treating Crohn’s disease, particularly in complex cases, by improving fistula closure rates and suggesting potential as a supplementary therapy. Its safety profile aligns with conventional treatments, yet ongoing clinical trials are crucial to optimize its use. Continual research will enable healthcare providers to tailor more effective treatment strategies for this challenging condition.

## Introduction

Highlights

- This umbrella review synthesizes evidence from 16 systematic reviews on the safety and efficacy of stem cell therapy for Crohn’s disease.
- Stem cell therapy increases the likelihood of clinical remission by 29.9% and healing perianal Crohn’s disease by 35.8% compared to conventional treatments.
- No significant impact was found on Crohn’s Disease Activity Index (CDAI) and Perianal Disease Activity Index (PDAI) scores.
- The safety profile of stem cell therapy is comparable to conventional therapies, with no significant increase in adverse or serious adverse events.
- Stem cell therapy shows promise for refractory Crohn’s disease cases, but further research and large-scale clinical trials are needed to conclusively determine its efficacy.

Crohn’s disease is a chronic, relapsing form of inflammatory bowel disease (IBD) characterized by the gastrointestinal tract inflammation^1^. The disease can affect any part of the gastrointestinal tract from the mouth to the anus, although it most commonly impacts the terminal ileum and colon. Patients with Crohn’s disease experience a wide range of debilitating symptoms, including diarrhea, abdominal pain, rectal bleeding, weight loss, and fatigue^1^. The etiology of Crohn’s disease remains elusive, though a multifactorial model incorporating genetic predisposition, environmental triggers, gut microbiota dysbiosis, and an aberrant immune response is increasingly recognized^2–4^.

Even with substantial progress in the therapeutic landscape of Crohn’s disease, many patients fail to achieve sustained remission with currently available therapies^5–7^. Conventional treatment options, including corticosteroids, immunomodulators (such as azathioprine and methotrexate), and biologic agents (like anti-TNF therapies), aim to suppress the inflammatory response and induce remission^8–10^. However, these treatments are associated with various limitations, including adverse effects, loss of response over time, and high costs. The unmet medical need for more effective and durable treatments has driven investigations into novel therapeutic approaches for Crohn’s disease, with stem cell therapy emerging as a promising area of research. Stem cell therapy harnesses the unique biological properties of stem cells, including their ability for self-renewal and multilineage differentiation potential, and their capability to modulate the immune system through paracrine effects^11–15^.

Several types of stem cell therapy has emerged as a promising avenue for the treatment of Crohn’s disease, including hematopoietic stem cells (HSCs), mesenchymal stem cells (MSCs), and more recently, induced pluripotent stem cells (iPSCs)^16^. MSCs isolated from various sources, including bone marrow, adipose tissue, and umbilical cord, have been the most extensively studied due to their immunomodulatory and tissue repair capabilities^17^. These mesenchymal MSCs exhibit a remarkable ability to home to sites of tissue damage and inflammation. There, they promote healing through a combination of mechanisms, including the secretion of anti-inflammatory and proregenerative factors, as well as direct interactions with resident cells^18,19^. Preclinical studies in animal models of IBD have provided encouraging evidence for the therapeutic potential of stem cell therapy^20–22^. MSCs have been shown to attenuate intestinal inflammation, promote mucosal healing, and modulate the immune response in these models. However, translating these promising findings into clinical practice has been challenging, with human trials yielding mixed results^20,23–26^.

Several systematic reviews have been conducted to synthesize the available evidence from clinical studies investigating the safety and efficacy of stem cells for treating Crohn’s disease^23,27,28^. These systematic reviews have focused on specific types of stem cells, such as MSCs or HSCs, or have evaluated stem cell therapy in the broader context of IBD, including both Crohn’s disease and ulcerative colitis. An umbrella review, which systematically synthesizes the findings from multiple systematic reviews, offers a unique opportunity to provide a comprehensive and up-to-date assessment of the efficacy of stem cell therapy for Crohn’s disease. By critically appraising and comparing the results of existing systematic reviews, an umbrella review can identify areas of consensus, highlight discrepancies, and pinpoint gaps in the current knowledge base.

This umbrella review aimed to identify and evaluate all systematic reviews examining the efficacy and safety of stem cell therapy for treating Crohn’s disease. By addressing these objectives, this umbrella review will provide a comprehensive evaluation of the current state of evidence regarding the efficacy of stem cell therapy for Crohn’s disease. The findings will inform clinical decision-making, guide the design and conduct of future clinical trials, and ultimately contribute to developing more effective and personalized treatment strategies for patients with this debilitating condition.

## Methods

This umbrella review was performed according to the Joanna Briggs Institute (JBI) methodology for umbrella reviews^29,30^. We followed the Preferred Reporting Items for Systematic Reviews and Meta-Analyses (PRISMA) guidelines for reporting the study (Table S1, Supplemental Digital Content 1, http://links.lww.com/JS9/D497)^31^. The protocol was registered with PROSPERO.

### Eligibility criteria

For this umbrella review, we strictly included only systematic reviews, whether they incorporated meta-analyses or not. The population of interest encompassed patients of any age who had been diagnosed with Crohn’s disease. We focused on interventions involving any type of stem cell therapy. This included MSCs sourced from various origins such as bone marrow, adipose tissue, and umbilical cord, as well as HSCs, induced iPSCs, and other types of stem cells or their derived products. The comparators considered in the eligible studies included placebo or sham interventions, standard medical therapy for Crohn’s disease, no treatment, and other active comparators. Our primary outcome of interest was clinical efficacy, which could be indicated by induction of remission, healing of fistula, or reduction in disease activity scores. Secondary outcomes focused on safety (adverse events and serious adverse events) and mortality rates. We considered only peer-reviewed published articles. Only articles available in English were included. We excluded systematic reviews that focused solely on ulcerative colitis or other forms of inflammatory bowel disease without specific reporting on Crohn’s disease, as well as reviews assessing stem cell therapy for nongastrointestinal diseases. Narrative reviews, editorials, commentaries, and other nonsystematic types of publications were excluded.

### Literature search

A comprehensive search of the literature was conducted across several databases, including Web of Science, Embase, PubMed, and the Cochrane Library, covering all records up to 20 April 2024. The search terms were strategically combined using Boolean operators (AND, OR) and included phrases like ‘Crohn’s disease’, ‘inflammatory bowel disease’, and ‘stem cell therapy’, along with keywords pertaining to systematic reviews and meta-analyses. The search was unrestricted by article type or language (Table S2, Supplemental Digital Content 1, http://links.lww.com/JS9/D497).

### Screening process

The study selection was facilitated by Nested-Knowledge software, which was used to eliminate duplicates before screening. Duplicate records were automatically removed with Nested-Knowledge. The screening was independently conducted by two researchers to reduce bias. They first reviewed titles and abstracts to ensure they met the inclusion criteria, followed by a thorough assessment of full texts for those deemed potentially relevant. Any disagreements were arbitrated by a third reviewer.

### Data extraction and quality assessment

Data from the qualified systematic reviews were meticulously extracted, including details such as author, databases included in the review, number and year of randomized controlled trials (RCTs) covered, types of interventions, outcomes evaluated, tools for risk of bias assessment, results of bias evaluation, and noted publication biases. This extraction utilized the ‘tagging’ feature of Nested Knowledge software, ensuring uniformity through a standardized form. The quality of the included reviews was appraised using the AMSTAR-2 tool.

### Data synthesis

Data were aggregated and analyzed for each specific outcome through meta-analyses conducted in R statistical software, version 4.3^32,33^. Outcomes were analyzed separately, with dichotomous outcomes yielding risk ratios (RR) and their CI by pooling event numbers and participant counts from the intervention and control groups. Continuous outcomes used means and SD. Heterogeneity was assessed using the *I*² statistic and τ², and a prediction interval was calculated. Significance was established at a *P*-value of less than 0.05.

## Results

### Literature search

**Figure 1:**
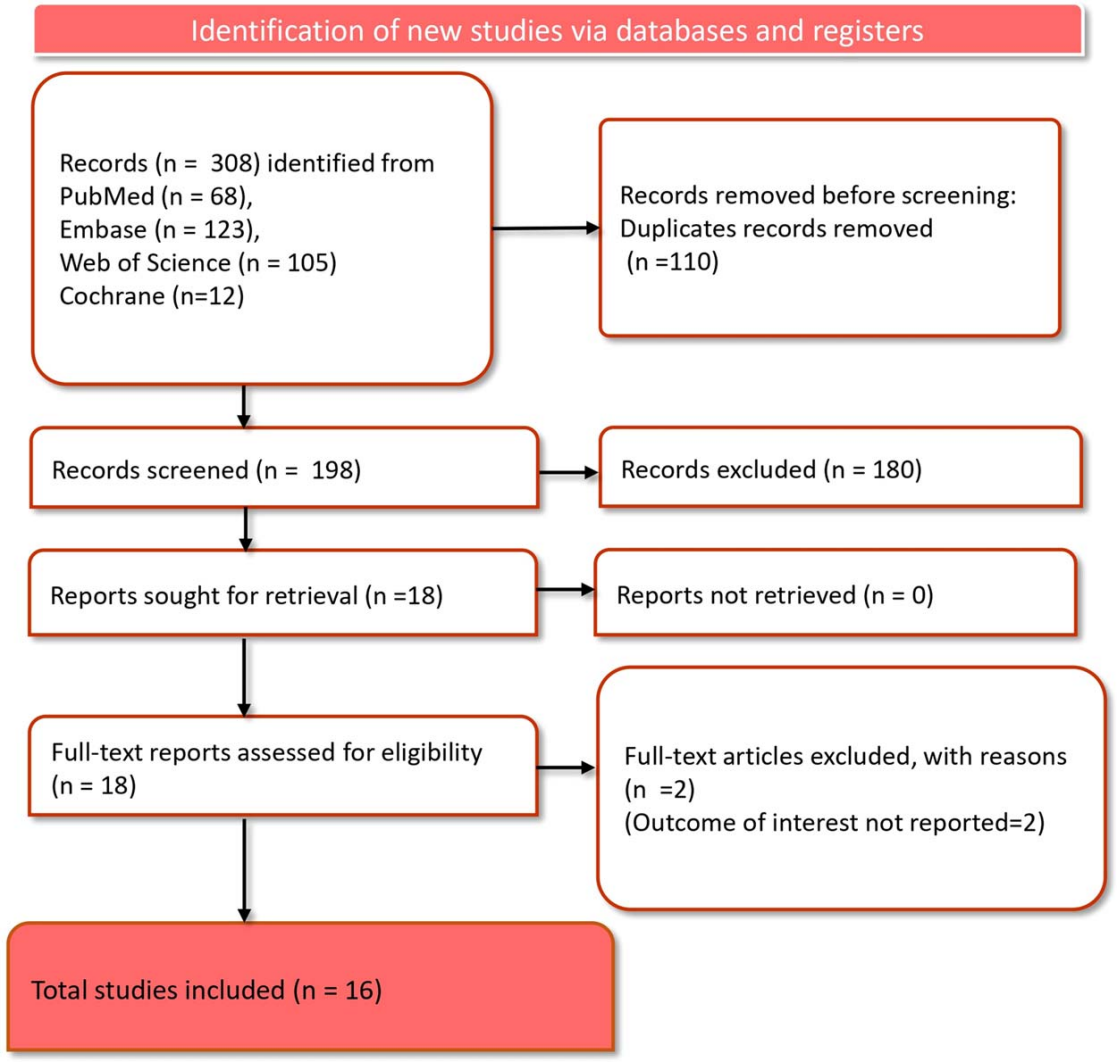
PRISMA flow diagram depicting article selection and screening process.

Initially, a total of 308 records were identified from multiple databases: 68 from PubMed, 123 from Embase, 105 from Web of Science, and 12 from Cochrane Library. Prior to the screening process, 110 duplicate records were removed, leaving 198 records to be screened. Of these, 180 records were excluded based on screening criteria, resulting in 18 reports that were sought for detailed evaluation. All 18 reports were retrieved, and full texts were assessed for eligibility. During this phase, two full-text articles were excluded because they did not report on the outcome of interest. Finally, 16 studies^28,34–48^ were included in the final analysis of the umbrella review (Fig. 1).

### Characteristics of included studies

The included systematic reviews span studies conducted between 2009 and 2023, utilizing major databases like PubMed, Embase, and the Cochrane Library, among others. The number of RCTs reviewed ranges from three to 29, indicating substantial research data analyzed across these reviews. The primary focus of these reviews is on adult patients diagnosed with Crohn’s disease, particularly those experiencing perianal fistulas and medically refractory conditions. Various types of stem cells are investigated, including autologous and allogeneic MSCs sourced from adipose tissue, bone marrow, placental tissue, and umbilical cords. Controls in these studies typically include placebo groups, no treatment, standard care, and other specific medical treatments like fibrin glue. The outcomes primarily monitored are clinical remission and fistula healing rates, alongside safety profiles, which include monitoring for adverse events and various clinical assessment scores like the Crohn’s Disease Activity Index (CDAI)(Table 1).

**Table 1:** Characteristics of the included reviews.

Study ID	Year and range of included studies	Databases and search	Number of included RCTs	Population	Type of stem cells	Type of control	Outcomes	Risk of bias of included RCTs	Risk of bias tool used	Publication bias	Key findings
Bernardi 2019^25^	2009–2018	PubMed and ScienceDirect, January 2008 to December 2018	13	Individuals with refractory Crohn’s disease	Autologous, allogeneic MSCs	Placebo	NA	Not assessed	Not assessed	Not assessed	MSCs are promising; more investigation is needed for luminal region transplant
Cao 2017^26^	2009–2016	PubMed, Web of Science, up to 30 September 2016	14	Patients diagnosed with CD	ASCs, BM-MSCs.	Placebo or standard of care	Overall healing rate	Low to moderate	NOS	Not assessed	MSCs are effective for Crohn’s fistula; CDAI baseline is a candidate for evaluating MSCs effectiveness
Cao 2021^27^	2009–2020	PubMed and other databases (Cochrane Library, Embase) from June 2005 to August 2020	29	Patients diagnosed with CD or IBD	ADSC, ADSC/SVF, BM-MSC, MSC	Placebo, standard of care	Fistula healing rate, treatment-related adverse events (TRAEs), CDAI scores, PDAI scores, IBDQ scores, and CRP levels	Moderate to high	NOS	Not assessed	Stem cells show promise for Crohn’s fistula treatment with high efficacy and low adverse events; more studies needed
Cheng 2019^28^	2009–2018	PubMed, Cochrane Library, Embase, and CNKI, from inception to October 2018	13	Patients diagnosed with CD perianal fistula by accepted criteria, no age or sex limits	MSCs	Placebo or standard of care	Healed perianal fistulas, clinical response, size of fistulas improvement, adverse events	Moderate to high	ROB 1	Not assessed	Local MSC administration is effective and safe for pCD; hope for treating refractory pCD
Cheng 2020^29^	2009–2019	PubMed and Embase, March 2020	7	Patients with complex perianal fistulas (either of cryptoglandular origin or associated with CD)	Autologous ASCs, Allogeneic ASCs	Fibrin glue or saline solution	Reepithelialization, ASCs Reepithelialization + MRI	Moderate to high	ROB 1	Not assessed	Local MSC therapy, alone or with fibrin glue, is safe and effective for complex perianal fistula
Cheng 2023^30^	2009–2022	PubMed, Embase, and Cochrane Library, March 2022	6	Patients with pCD aged ≥18 years	Autologous or allogeneic MSC	Placebo, standard of care	Reepithelialization, Reepithelialization + MRI	Low to moderate	ROB 1	The funnel plot does not indicate publication bias	Local MSC injection is safe and effective for perianal fistulas in CD, with favorable long-term efficacy and safety profiles
Ciccocioppo 2019^31^	2009–2018	PubMed, EMBASE, Web of Science, Cochrane databases, CINAHL, and ClinicalTrials.gov, May 15, 2017	15	Patients with perianal CD	Autologous MSC, Allogeneic MSC.	Placebo/comparator or standard of care	Safety (AE, related AE, severe related AE, death, hospitalization), efficacy (external healing, radiological healing, endoscopic healing, combined end-point, CDAI, PDAI, fistula recurrence)	Moderate to high	ROB 1	No publication bias evidence from funnel plots	Local MSC injection is safe and effective
Dave 2015^32^	2009–2013	PubMed (since inception to March 2015) and EMBASE (since inception to November 2014)	6	Patients with IBD	Allogenic BM MSC, autologous BM MSC	Placebo or standard of care	Healing of perianal fistulas, clinical remission	High risk	ROB 1	Not assessed	MSCs are an alternative treatment for refractory IBD; safe and potentially effective, but more studies needed
El-Nakeep 2022^33^	2009–2020	Cochrane Central Register of Controlled Trials (CENTRAL), Embase	7	Participants with refractory Crohn’s disease who received one or more failed standard treatments	Autologous, allogeneic stem cells	Placebo, standard of care	Clinical remission, fistula closure short-term and long-term, total adverse events, serious adverse events, withdrawal due to adverse events	Low to moderate	ROB 1	Not assessed	Systemic stem cell therapy’s benefit for clinical remission is uncertain; local injection may help fistula closure
Ko 2021^34^	2009–2020	PubMed from inception to 29 October 2020	4	Patients with IBD	MSC	Normal saline (placebo)	Decrease in CDAI, total Mayo UC activity score, remission rate and average remission duration, decrease in CDAI, HBI, and corticosteroid usage	Not assessed	Not assessed	Not assessed	MSCs show potential for IBD treatment due to their immunomodulatory effects; robust evidence for local injections in PFCD
Li 2023^35^	2016–2022	PubMed, Cochrane Library, Embase	5	Patients diagnosed with CD perianal fistula	MSCs	Placebo	Remission, TEAEs, perianal abscess, and proctalgia.	Moderate to high	ROB 1	Presence of publication bias	MSCs are effective and safe for pfCD, with the potential for combination with traditional therapies
Lightner 2018^36^	2009–2016	PubMed, Cochrane Library Central Register of Controlled Trials, and Embase	3	Refractory perianal Crohn’s disease	MSCs	Placebo or standard of care	Adverse events, SAE, healing rate	Moderate to high	ROB 1	Not assessed	MSCs show improved efficacy and no increase in adverse events for treating perianal Crohn’s disease
Qiu 2017^37^	2009–2016	PubMed, Cochrane Library CENTRAL, and Embase (initial search 5 February 2015; updated 15 October 2016)	6	Patients diagnosed with CD by accepted criteria, not in clinical or endoscopic remission at study’s outset	HSC, BMSC, ASC	Placebo or standard of care	Endoscopic remission, clinical remission, systemic infusion for CD, clinical response	Moderate to high	ROB 1	Egger test suggests no publication bias for fistula healing but exists for clinical response	SCT may be an alternative treatment for refractory active CD; toxicity is a barrier to systemic SCT
Qiu 2024^38^	2009–2023	Medline (PubMed), CENTER (Cochrane Library), and Embase (Ovid), 05 September 2023	12	Adult patients with medically refractory CD or CD-related fistula	ADSCs, autologous BM-MSCs, allogeneic placental-derived cells, allogeneic HSCs, autologous adipose-derived MSCs, allogenic UC-MSCs, allogenic	Placebo or no treatment	Clinical remission (CR) and severe adverse events (SAE)	Moderate to high	ROB 2	The funnel plot indicated the minimal risk of publication bias	SCT may improve CR in medically resistant CD or CD-associated fistula without increasing SAE risk. Further research needed for long-term effectiveness and safety
Wang 2021^39^	2015–2020	PubMed, Embase, Web of Science, Cochrane Library, and Clinical Trials.gov, from inception to February 2021	18	Patients with CD	Allogeneic MSC, Autologous HSC, Autologous BM-MSC, PDA-001, Allogeneic BM-MSC	Placebo, standard of care	CD clinical parameters (CDAI scores, CRP levels, CD-EIS scores, SES-CD, IBDQ results), histopathological scores (HSs), colon lengths, MPO, and cytokine levels	Moderate to high	MINORS tool	Potential publication bias was found	SC therapy improves CD by reducing gut inflammation and enhancing the quality of life; more high-quality trials are needed
Wang 2023^40^	2009–2022	PubMed, Embase, Cochrane Library, and US ClinicalTrials.gov, 15 May 2022	10	The perianal fistulas of every participant were of cryptoglandular origin or associated with CD	ASCs autologous, BSCs allogenic, ASCs allogenic	First: fibrin glue, Second: double dose	Reepithelialization + MRI/ERUS	Low to moderate	ROB 1	The funnel plot showed that no publication bias existed	MSC transplantation is effective for complex perianal fistulas in both origins, showing high efficacy in the short and long term

ADSC, adipose-derived stem cells; AE, adverse event; ASC, adipose-derived stromal cells; BM-MSC, Bone marrow-derived mesenchymal stem cells; CD, Crohn’s disease; CDAI, Crohn’s disease activity index; CD-EIS, Crohn’s disease endoscopic index of severity; CR, clinical remission; CRP, c-reactive protein; GRADE, grading of recommendations assessment, development and evaluation; HBI, Harvey-Bradshaw index; HSC, hematopoietic stem cells; IBD, inflammatory bowel disease; IBDQ, inflammatory bowel disease questionnaire; MINORS, methodological index for non-randomized studies; MPO, myeloperoxidase; MSC, mesenchymal stem cell; NA, not available; NOS, Newcastle–Ottawa Scale; PDA-001, cell-based therapy product; PDAI, perianal disease activity index; RCT, randomized controlled trial; ROB, risk of bias; SAE, serious adverse event; SCT, stem cell therapy; SES-CD, simple endoscopic score for Crohn’s disease; SVF, stromal vascular fraction; TEAE, treatment-emergent adverse event; TRAE, treatment-related adverse event; UC, ulcerative colitis; UC-MSC, umbilical cord-derived mesenchymal stem cells.

Methodological considerations reveal that most studies report a moderate to high risk of bias, assessed using tools like ROB 1, ROB 2, and the Newcastle–Ottawa Scale (NOS). Some studies assess the certainty of evidence using the GRADE approach, often finding it to be of moderate certainty. Publication bias is evaluated mostly through funnel plots, with several studies reporting minimal risk, although some do note potential biases. Key findings across these reviews suggest that stem cell therapies, particularly involving MSCs, are effective and safe in treating Crohn’s disease-associated fistulas and other symptoms. The assessment of study quality is given in Table S3 (Supplemental Digital Content 1, http://links.lww.com/JS9/D497).

### Clinical remission

**Figure 2:**
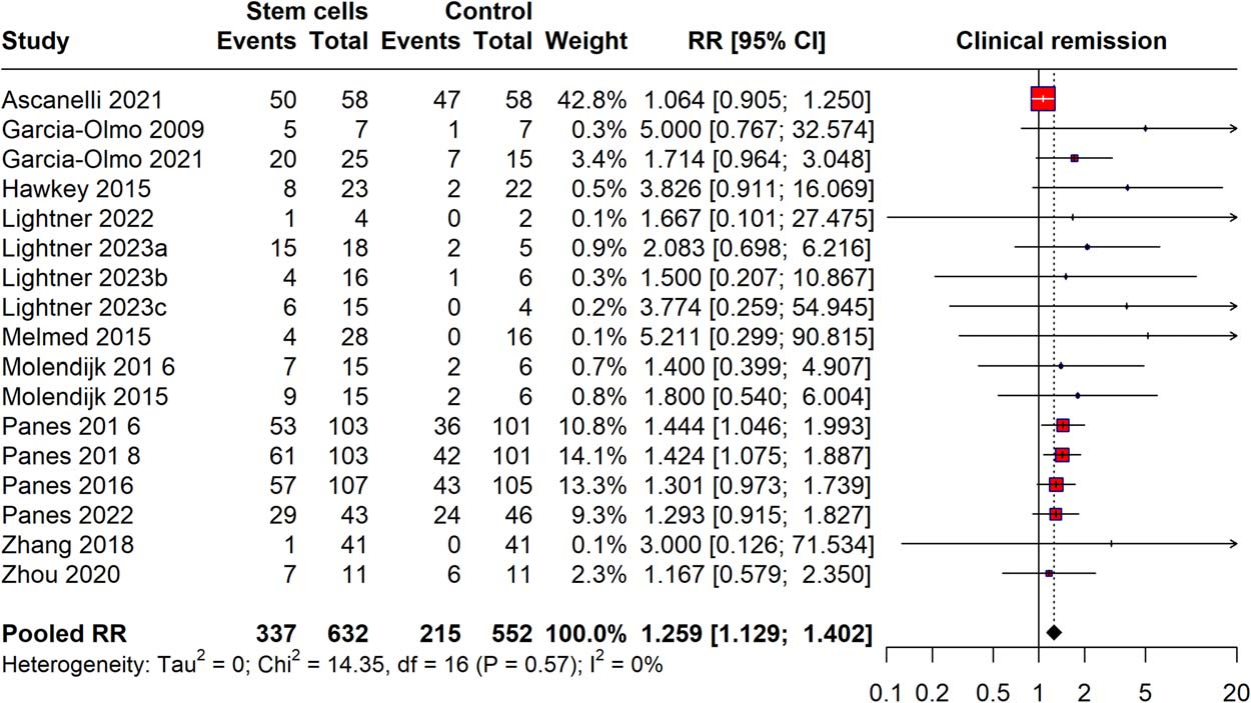
Clinical remission of Chron’s disease with stem cell therapy.

The meta-analysis evaluated the efficacy of stem cell therapy for inducing clinical remission in Crohn’s disease patients, comparing data from studies conducted between 2003 and 2023. The results show a pooled RR of 1.299 (95% CI: 1.192–1.420), indicating that stem cell therapy is associated with a 29.9% increased likelihood of achieving clinical remission compared to controls. The analysis reveals very low heterogeneity (*I*²=0%), suggesting consistent effects across the included studies. This suggests that stem cell therapy may effectively induce clinical remission in Crohn’s patients (Fig. 2).

### Healing of perianal Crohn’s disease

**Figure 3:**
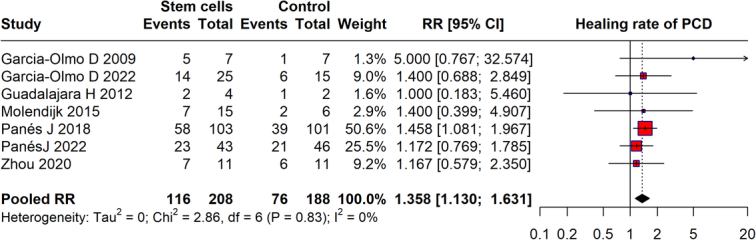
Healing rate of perianal Chron’s disease with stem cell therapy.

The meta-analysis examined the healing rates of perianal Crohn’s disease (PCD) using stem cell therapy, incorporating data from studies (Fig. 3). The pooled RR of 1.358 (95% CI: 1.13–1.631) indicates that stem cell therapy is associated with a 35.8% increased likelihood of healing in PCD compared to controls. The analysis also shows very low heterogeneity (*I*²=0%), suggesting consistent effects of stem cell treatment across different studies. This indicates that stem cell therapy may effectively enhance healing rates in PCD patients.

### CDAI score

**Figure 4:**
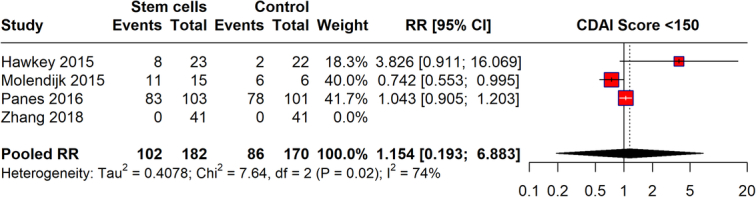
CDAI score <150 with stem cell therapy.

The meta-analysis assessed the effectiveness of stem cells in achieving a CDAI score of less than 150, using data from studies conducted between 2015 and 2018 (Fig. 4). The pooled RR from the meta-analysis is 1.154 (95% CI: 0.193–6.883), suggesting no significant effect. However, the heterogeneity among the studies is relatively high (*I*²=74%), indicating variability in the treatment effects across studies. This suggests that the effectiveness can vary significantly between different settings or study designs.

### PDAI score

**Figure 5:**
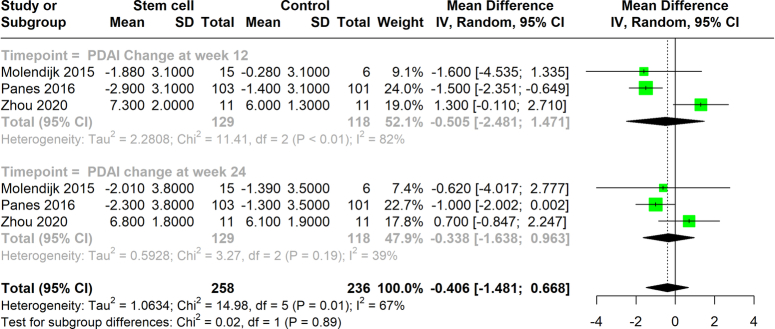
Change in PDAI Scores at week 12 and week 24 with stem cell therapy.

We evaluated the impact of stem cell therapy on mean PDAI scores in patients, comparing stem cell treatment groups with controls. The analysis found that the change in PDAI scores at 12 weeks showed a mean difference of −0.505 (95% CI: −2.481 to 1.471), with the heterogeneity of *I*²=82%. At 24 weeks, the meta-analysis revealed a mean difference of −0.338 (95% CI: −1.638 to 0.963), with heterogeneity of *I*²=39%. These results indicate no significant effect of stem cell therapy on reducing mean PDAI scores (Fig. 5).

### Fistula closure

**Figure 6:**
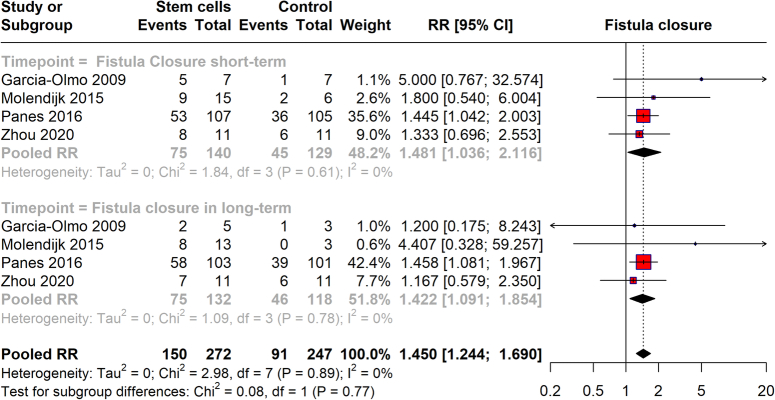
Fistula closure with stem cell therapy.

The meta-analysis evaluated the efficacy of stem cell treatment for achieving fistula closure in patients with Crohn’s disease, with data categorized into long-term and short-term outcomes (Fig. 6). For short-term outcomes, the analysis demonstrated a pooled RR of 1.481 (95% CI: 1.036–2.116). This suggests a 48.1% higher likelihood of achieving fistula closure shortly after treatment than controls with very low heterogeneity observed across the studies (*I*²=0%). In terms of long-term outcomes, the same set of studies provided a pooled RR of 1.422 (95% CI: 1.091–1.854), indicating a continued favorable outcome with a 42.2% greater likelihood of maintaining fistula closure over the long term with low heterogeneity (*I*²=0%). This meta-analysis robustly supports the use of stem cell therapy as a beneficial treatment for fistula closure in Crohn’s disease patients, consistently across both short and long-term periods.

### All-cause mortality

**Figure 7:**
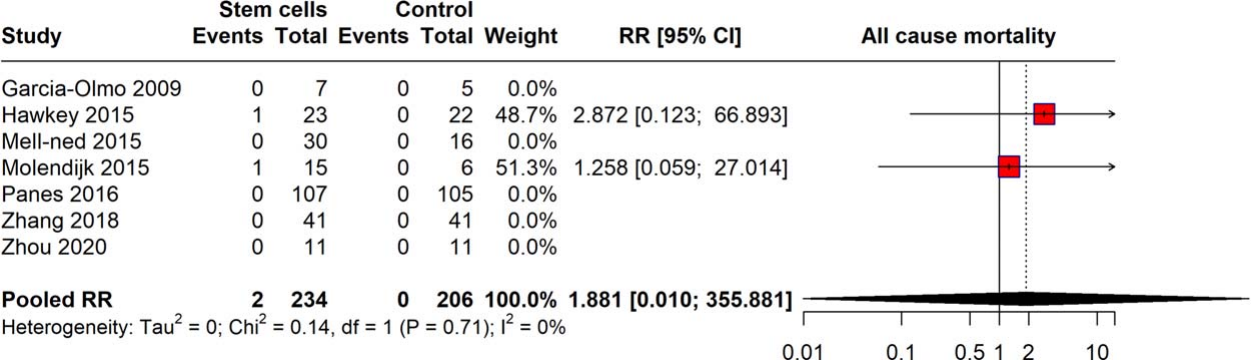
All-cause mortality with stem cell therapy compared to control group.

The meta-analysis evaluated the impact of stem cell therapy on all-cause mortality compared to controls (Fig. 7). The pooled RR calculated for all-cause mortality was 1.881 (95% CI: 0.010–355.881), reflecting the small number of mortality events—two in stem cell groups and none in controls among 234 and 206 participants, respectively. Despite the zero heterogeneity (*I*²=0%), indicating consistent results across studies, the wide CI greatly undermines the reliability of this estimate. This analysis suggests a potentially higher RR of mortality with stem cell therapy, but the actual risk remains indeterminate due to limited events and significant statistical uncertainty. Further research with larger sample sizes is required to provide a clearer understanding of the mortality risks associated with stem cell therapy.

### Adverse events

**Figure 8:**
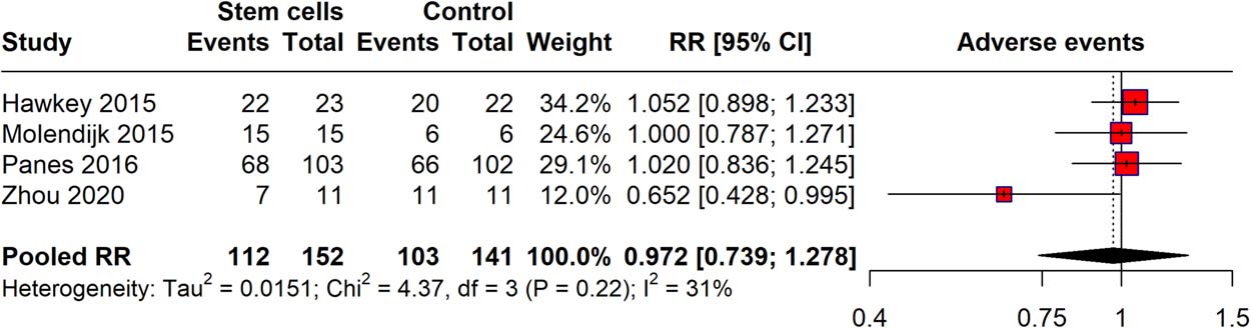
Adverse events in with stem cell therapy.

The meta-analysis evaluated the risk of adverse events associated with stem cell therapy compared to controls in various studies. The plot shows the number of adverse events recorded in both stem cell and control groups, with the total number of events being 112 in the stem cell group and 103 in the control group, from a total of 152 and 141 participants, respectively (Fig. 8). The pooled RR for adverse events from stem cell therapy is 0.972 (95% CI: 0.739–1.278). This indicates that there is no significant difference in the risk of adverse events between those receiving stem cell treatments and controls, as the confidence interval includes the value 1. The heterogeneity among the studies is relatively low (*I*²=31%), suggesting moderate variability in the results across the included studies.

### Serious adverse events

**Figure 9:**
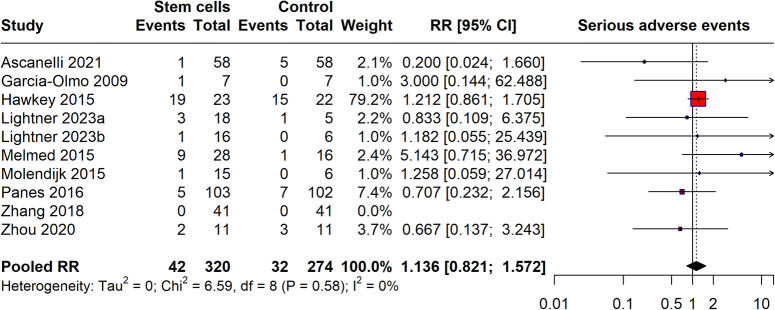
Serious adverse events.

The meta-analysis evaluates the risk of serious adverse events (SAEs) associated with stem cell therapy compared to controls across several studies (Fig. 9). The analysis finds that the stem cell groups experienced 42 SAEs among 320 participants, while the control groups reported 32 SAEs out of 274 participants, resulting in a pooled RR of 1.136 (95% CI: 0.821–1.572). This CI crosses 1, indicating that the slightly higher risk of SAEs in the stem cell groups is not statistically significant. The heterogeneity across the studies is very low (*I*²=0%), suggesting consistent results and confirming that stem cell therapy does not significantly increase the risk of serious adverse outcomes compared to controls.

### Withdrawal due to adverse events

**Figure 10:**
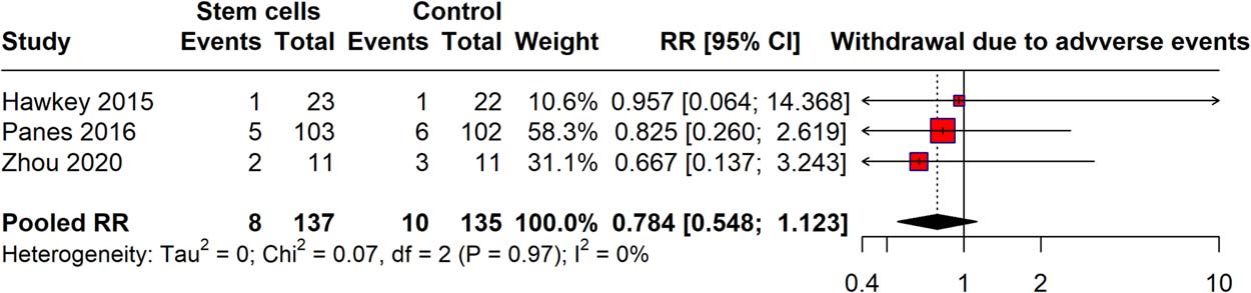
Withdrawal due to adverse event.

The meta-analysis investigated withdrawal rates due to adverse events between the stem cell therapy arm and the control arm across studies. It reveals that there were eight withdrawals due to adverse events out of 137 patients in the stem cell group, and 10 out of 135 in the control groups. The pooled RR is calculated at 0.784 (95% CI: 0.548–1.123), indicating that the difference in withdrawal rates due to adverse events between the stem cell therapy and control groups is not statistically significant. The very low heterogeneity (*I*²=0%) across the studies suggests a consistent pattern in the findings, and the analysis concludes that stem cell therapy does not significantly increase the likelihood of study withdrawal due to adverse events compared to conventional treatments, pointing to a generally comparable safety profile between the two (Fig. 10).

## Discussion

This umbrella review synthesizes multiple systematic reviews to evaluate the efficacy of stem cell therapy in treating Crohn’s disease, a chronic and often debilitating form of IBD. Despite advances in medical treatments, achieving sustained remission in Crohn’s disease remains a significant challenge, propelling the exploration of alternative therapies like stem cell interventions. Our analysis revealed a promising potential for stem cell therapy to induce clinical remission and heal perianal Crohn’s disease. With a pooled RR of 1.299 for clinical remission, stem cell therapy shows a nearly 30% greater likelihood of remission compared to conventional therapies. Similarly, the healing rates for perianal Crohn’s disease demonstrated a pooled RR of 1.358, suggesting a 35.8% increased likelihood of healing. These findings suggest the therapy’s potential to significantly impact disease outcomes positively, especially for patients who have not responded to traditional treatments.

The effects of stem cell therapy on CDAI and PDAI scores, however, presented a more nuanced picture. While the therapy did not significantly impact CDAI scores, as evidenced by the high heterogeneity among the studies, it highlights the variability in treatment effectiveness. This emphasizes the influence of the source from which stem cells are derived. For PDAI scores, the minimal changes observed suggest that while stem cells may not drastically reduce disease activity scores, they may still play a role in managing specific symptoms or cases. One of the most compelling findings from our review is the efficacy of stem cell therapy in achieving fistula closure in Crohn’s disease. The short-term and long-term pooled RRs (1.481 and 1.422, respectively) indicate a robust potential for stem cells to promote healing and possibly sustain it over time. This is particularly significant for patients suffering from fistulating disease, who often experience considerable morbidity and limited treatment options.

The safety profile of stem cell therapy, as analyzed through adverse events and serious adverse events, reveals a reassuring aspect of this therapeutic approach. The pooled RR for adverse events was 0.972, indicating no significant increase in risk compared to controls. For serious adverse events, the risk was slightly higher but not statistically significant, with a pooled RR of 1.136. These outcomes suggest that while stem cell therapy is not devoid of risks, its safety profile is comparable to that of existing treatments. Concerning all-cause mortality and withdrawals due to adverse events, our findings provide critical insights into the risk management of stem cell therapy. The all-cause mortality rate, though potentially higher, showed a vast CI, reflecting the need for further research with larger sample sizes to ascertain the true mortality risk. Withdrawals due to adverse events were not significantly higher in the stem cell groups, reinforcing the manageable safety profile of this therapy.

The quality assessment of the included systematic reviews revealed varying levels of bias. Studies such as Cheng *et al*. 2023 and El-Nakeep *et al*. 2022 had lower appraisals due to unclear or absent assessments of publication bias and conflict of interest strategies. Moderate appraisals for Bernardi *et al*. 2019, Cao *et al*. 2021, and Qiu *et al*. 2017 were due to limitations in bias assessment and handling heterogeneity. High-quality studies like Dave *et al*. 2015 and Wang *et al*. 2021 consistently met most criteria, enhancing their reliability. This variability in study quality highlights the need for transparent reporting and robust methodologies in future reviews to optimize stem cell therapy protocols for Crohn’s disease.

Stem cell therapy has shown significant potential for achieving clinical remission and fistula healing in treatment-resistant Crohn’s disease, aligning with findings by El-Nakeep *et al*. 2022 and Qiu *et al*. 2020, who reported favorable outcomes with stem cell therapy in refractory Crohn’s disease. Specifically, stem cell therapy’s effectiveness in perianal fistulizing Crohn’s disease (PFCD) is corroborated by Ko *et al*. 2021, who emphasized its strong efficacy profile. Stem cell therapy demonstrated a 29.9% increased likelihood of clinical remission and a 35.8% increased likelihood of healing perianal Crohn’s disease. However, the variability in CDAI and PDAI scores, as noted in our review and supported by Dave *et al*. 2015, suggests the benefits of stem cell therapy may depend on specific applications and administration methods. Despite the promising results, Stem cell therapy’s effectiveness varies due to differences in stem cell types, sources, and administration methods, as seen in the mixed results for systemic infusions in luminal IBD. El-Nakeep *et al*. 2022 reported that Stem cell therapy is particularly promising for Crohn’s disease patients who have failed to respond to traditional treatments, emphasizing that Stem cell therapy’s favorable safety profile makes it a viable option. Ko *et al*. 2021 highlighted that local injections of MSCs have been especially effective for PFCD, which aligns with our findings of improved fistula closure rates. Dave *et al*. 2015 also noted that stem cell therapy does not significantly increase serious adverse events compared to conventional treatments. The necessity for standardized protocols is evident to ensure consistent outcomes across studies.

The findings suggest the potential of stem cells as a promising therapeutic alternative for patients who struggle to achieve remission through traditional treatment methods. The evidence supports stem cell therapy’s potential in inducing clinical remission and healing perianal Crohn’s disease, areas where conventional treatments often fall short. Patients who fail to respond to conventional therapies like 5-ASA, corticosteroids, and purine analogs are typically treated with anti-TNF agents like infliximab and adalimumab. However, the efficacy of anti-TNF agents is limited due to primary nonresponse or loss of response over time. In these cases, options include switching to another anti-TNF agent, combining anti-TNF therapy with azathioprine, or using granulocyte and monocyte adsorptive apheresis. For clinicians, stem cell therapy can be an additional tool that could significantly improve outcomes for patients with severe or refractory Crohn’s disease. However, implementing this therapy should proceed with caution, careful patient selection, and close monitoring, which are crucial due to the variable efficacy and safety profile indicated by the reviewed studies.

As this field evolves, clinical guidelines need to be updated to incorporate stem cell therapy as a viable option, particularly for patients who have exhausted other treatments, ensuring that practice keeps pace with emerging evidence. Future research should focus on large-scale RCTs to explore the long-term effects and safety of stem cell therapies in diverse patient populations. Additionally, head-to-head trials comparing different sources of stem cells and their administration protocols are necessary to optimize treatment outcomes. Investigating the mechanisms of action behind stem cells’ immunomodulatory and regenerative capacities is of significant interest in therapeutic applications. This includes examining the immunomodulatory crosstalk between stem cells and the immune system and identifying the most effective cell types and delivery methods. Expanding the research to include diverse patient demographics and disease severities will provide more comprehensive data, ultimately guiding the development of personalized treatment strategies. Continued research efforts are essential to establish standardized protocols, ensure consistent therapeutic results, and integrate stem cell therapy into mainstream clinical practice for Crohn’s disease.

While this umbrella review provides valuable insights into the safety and efficacy of stem cells for Crohn’s disease, several limitations must be acknowledged. First, the inherent variability in the types of stem cells used (e.g. MSCs, HSCs, and iPSCs), their sources (adipose tissue, bone marrow, and umbilical cord), and administration protocols across studies introduces heterogeneity that can affect the consistency of the findings. Second, many of the included systematic reviews and meta-analyses have a moderate to high risk of bias, which may influence the reliability of the conclusions. Some studies’ relatively small sample sizes can also restrict the study’s statistical power to identify statistically significant differences between groups, particularly in adverse events and mortality outcomes. Additionally, the follow-up periods varied significantly among studies, making it challenging to comprehensively assess the long-term efficacy and safety of stem cell therapies. The generalizability of the findings is limited by the lack of standardized protocols and the diverse patient populations included in the studies, which may not fully represent the broader Crohn’s disease patient community. We only included studies available in the English language. Addressing these limitations in future research is crucial for validating the therapeutic potential of stem cells.

## Conclusion

Stem cell therapy represents a significant advancement in treating Crohn’s disease, offering hope for clinical remission and healing, especially in complex cases. It has shown improvements in fistula closure rates, suggesting its potential as an adjunct to other therapies. While its safety profile is comparable to conventional therapies, ongoing clinical trials are essential to fully realize its potential and integrate it effectively into clinical practice. By building on the current knowledge base, healthcare providers can better strategize and personalize treatments to enhance outcomes for patients with this challenging condition

## Ethical approval

Not applicable.

## Consent

All authors gave consent for publication.

## Source of funding

This study received no funding.

## Author contribution

L.T., S.M., M.N.K., Q.S.Z., S.I.A., G.B., and M.S.: conceptualization; H.A.A., A.F., and S.V.M.: data curation; P.B., C.C., M.R.K., and R.V.: formal analysis; A.M.A.A.-R., S.R., A.K.M., and S.H.A.A.: investigation; M.N.K., Q.S.Z., S.I.A., and G.B.: methodology; S.R., S.H.A.A., H.A.A., and A.A.R.: project administration; L.T., S.M., and S.V.M.: resources; M.N.K., Q.S.Z., S.I.A., and G.B.: software; S.M., M.N.K., Q.S.Z., S.I.A., G.B.: supervision; H.A.A., A.F., S.V.M., and M.S.: validation; R.V., A.M.A.A.-R., H.A.A., and A.A.R.: visualization.

## Conflicts of interest disclosure

The authors declare no conflicts of interest.

## Research registration unique identifying number (UIN)

PROSPERO: CRD42024552345.

## Guarantor

Ganesh Bushi.

## Data availability statement

All data are presented within the manuscript and are available by contacting the corresponding author.

## Provenance and peer review

Not commissioned, externally peer-reviewed.

## Supplementary Material

SUPPLEMENTARY MATERIAL
